# Surgery-first approach with 3D customized passive self-ligating brackets and 3D surgical planning: Case report

**DOI:** 10.1590/2177-6709.23.3.047-057.oar

**Published:** 2018

**Authors:** Juan Fernando Aristizábal, Rosana Martínez-Smit, Cristian Díaz, Valfrido Antonio Pereira

**Affiliations:** 1 Universidad del Valle, Departamento de Ortodoncia (Cali, Colombia).; 2 CES University, Departamento de Ortodoncia (Medellín, Colombia).; 3 Universidade Estadual Paulista, Departamento de Ortodontia e Pediatria, Faculdade de Odontologia de Araraquara (Araraquara/SP, Brazil).; 4 Universidade Estadual Paulista, Departamento de Diagnóstico e Cirurgia Bucomaxilofacial, Faculdade de Odontologia de Araraquara (Araraquara/SP, Brazil).

**Keywords:** Angle Class III malocclusion, Orthodontic surgery, Orthodontics, Three-dimensional image

## Abstract

It is possible to unify three-dimensional customized orthodontic techniques and three-dimensional surgical technology. In this case report, it is introduced a treatment scheme consisting of passive self-ligation customized brackets and virtual surgical planning combined with the orthognathic surgery-first approach in a Class III malocclusion patient. Excellent facial and occlusal outcomes were obtained in a reduced treatment time of five months.

## RESUMO

É possível unificar técnicas ortodônticas personalizadas e tecnologia de planejamento cirúrgico 3D. No presente relato de caso, apresenta-se um plano de tratamento envolvendo o uso de braquetes autoligáveis passivos personalizados e planejamento cirúrgico virtual, combinado com cirurgia ortognática de benefício antecipado, em um paciente com má oclusão de Classe III. Foram obtidos excelentes resultados faciais e oclusais em um tempo reduzido de tratamento, de 5 meses.

## INTRODUCTION

Accurate surgical treatment starts with precise diagnosis, by evaluating all dimensions and determining the nature of deformity, because it might be a combination of hard and soft tissue components.^1^

The main limitation of conventional surgical planning is its two-dimensional approach that involves clinical examination, extraoral and intraoral photographs, lateral and posteroanterior cephalograms, and plaster dental models.^2^ ^,^ ^3^ To overcome these deficiencies, cone-beam computed tomography (CBCT) for imaging the craniofacial region brought a true paradigm shift from a two-dimensional to a three-dimensional (3D) approach.^4^

Computer-aided surgical simulation (CASS) utilizing three-dimensional images obtained from multislice computed tomography (MSCT)/cone beam computer tomography (CBCT) has been successfully performed previously to plan craniofacial surgery.^5-8^ Also, CASS has been combined with the surgery-first approach (SFA) to demonstrate two useful and practical methods for planning these cases.^9^

Furthermore, the patient can be virtually visualized by generating a fusion model with digital dental casts, a CBCT reconstructed bony volume and textured facial soft tissue image.^10^ ^,^ ^11^ Additionally, with this fusion model the clinicians can accurately create surgical splints using the computer-aided design/computer-aided manufacturing (CAD/CAM) system for successful surgical treatments.^11^ ^,^ ^12^

Recently, significant technological advancements have been made in computer-aided orthodontic treatment. In the Insignia system (Ormco Corporation, Orange County, CA), polyvinyl siloxane (PVS) impressions are digitized with computed tomography to produce highly detailed digital models, or an intraoral dental scanner is used to generate 3D digital models. The orthodontist adjusts the digital setup using a real-time 3D interface, while referring to the patient’s intra and extraoral photographs and radiographs for consideration of esthetic treatment goals. After the clinician approves the final setup, the customized brackets, tubes, and arch-wires are fabricated and bracket-positioning jigs are provided, for accurate indirect transfer.^13^

In the present case report, 3D virtual customized bracket design (Insignia, Ormco Corporation, Orange County, CA) was integrated with 3D virtual surgical planning along with fabrication of digital surgical splints using a CAD/CAM technique. This article aims to report how the use of 3D digital technology, self-ligating brackets and the SFA can drastically reduce treatment time.

## CASE REPORT

**Figure 1:**
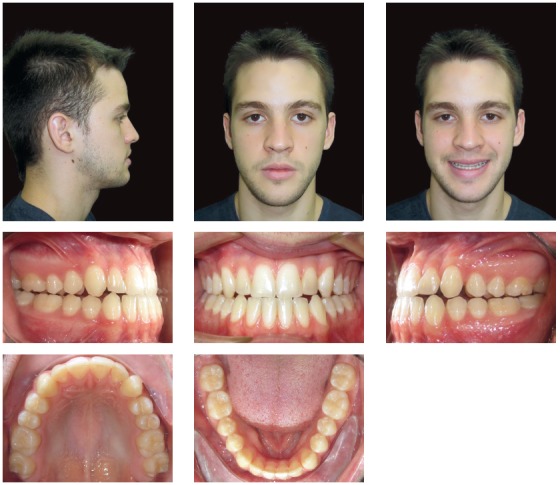
Pre-treatment photographs showing skeletal and dental Class III malocclusion.

A 21-year-old Hispanic male reported to the orthodontist office with the primary complaint of not feeling comfortable with the bite and chin projection (Fig 1). A subsequent clinical examination showed that the profile had worsened since a previous orthodontic treatment. 

**Figure 2:**
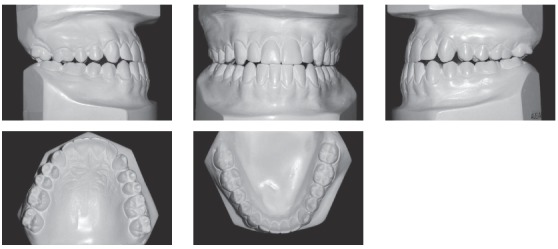
Pre-treatment dental casts.

**Figure 3:**
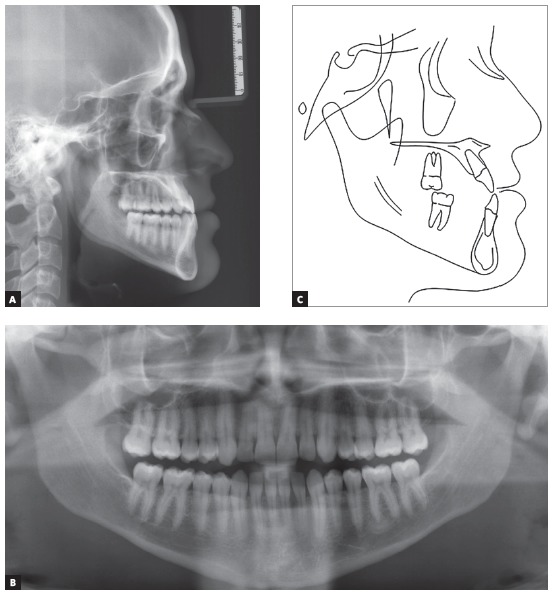
A) Pre-treatment lateral cephalometric radiograph. B) Pre-treatment panoramic radiograph. C) Pre-treatment lateral cephalometric tracing.

Systemically, he referred controlled Diabetes Mellitus Type I. The extraoral examination showed concave facial profile, with a slight maxillary hypoplasia, significant chin projection, upper lip retrusion and adequate nasolabial angle (Fig 1). Dentally, the patient presented a Class III malocclusion with proclined upper incisors and retroclined lower incisors, edge to edge bite, lower proper alignment and spacing of 2mm in the upper arch (Figs 1, 2, and 3A).The panoramic radiograph showed mild different ramus lengths (Fig 3B). Skeletally, Class III pattern with mandibular prognathism and macrognathism was observed (Fig 3A, 3C).

The treatment objectives were to correct the Class III skeletal pattern, to improve profile, to increase overjet and to improve facial aesthetics. The treatment options presented were presurgical orthodontic treatment followed by mandibular setback surgery and SFA with mandibular setback followed by fixed appliances to align, level and stabilize the occlusion. Considering that the patient’s chief concern was his facial esthetics, it was decided to proceed with SFA, because the patient wanted immediate facial change. This approach would avoid deterioration in his profile and malocclusion during presurgical orthodontics, and would also take advantage of the biological potential of the regional acceleratory phenomenon (RAP). 

**Figure 4:**
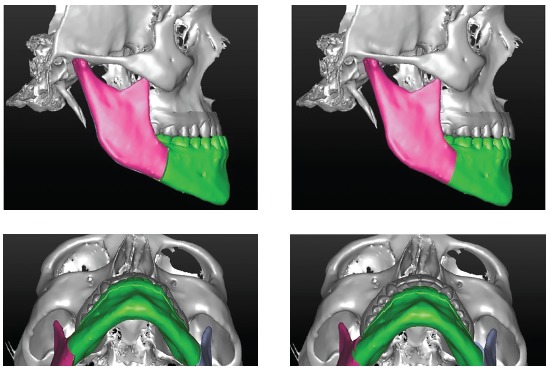
Surgical planning of mandibular setback.

A computed tomography (CT) (Bright Speed Elite, General Electric, and Fairfield, Connecticut, USA) was taken for the construction of a model of the skull^8^ with Proplan CMF (Materialise, Plymouth, MIs). The surgical plan was mandibular setback (Fig 4). The virtual design was transferred to the CAD/CAM software for production of surgical splints. The intermediate splint was physically generated by a 3D printer (Fortus 250mc, Stratasys, Eden Prairie, MN, USA) with hybrid epoxy-acrylate polymer.

**Figure 5:**
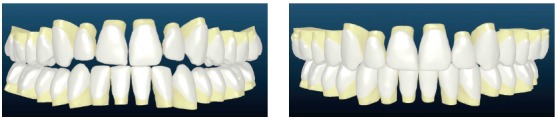
Custom designed orthodontics, with Insignia.

The first step in the Insignia system (Ormco Corporation, Orange, CA) for custom-designed orthodontics is to send precise polyvinyl siloxane impressions as well as photographic and radiographic information to the manufacturer. The brackets chosen were Insignia self-ligating (SL) brackets, which are the customized version of Damon Q SL brackets (Ormco Corporation, Orange, CA).^14^ The final set-up for the patient was approved with an overcorrection of lower incisors positive torque, ensuring optimal expression of the lower incisors decompensation exploiting the massive RAP after orthognathic surgery (Fig 5). The selected sequence of wires was CuNiTi 0.014-in, CuNiTi 0.014 x 0.025-in, CuNiTi 0.018 x 0.025-in, TMA 0.019 x 0.025-in and stainless steel 0.019 x 0.025-in (Ormco Corporation, Orange, CA). The brackets were bonded three days before surgery and no archwire was placed.

**Figure 6:**
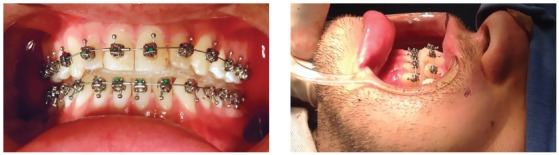
First archwire placed during the surgery.

In the day of the surgery, immediately before intubation assisted by a fiber optic probe, CuNiTi 0.014-in (Ormco Corporation, Orange, CA) archwires were placed (Fig 6). After mandibular setback surgery by sagittal osteotomy, under brain activity monitoring, and once a suitable rigid fixation and postoperative occlusion were established, ¼ 3.5 oz intermaxillary elastics were applied with Class III vector. 

**Figure 7:**
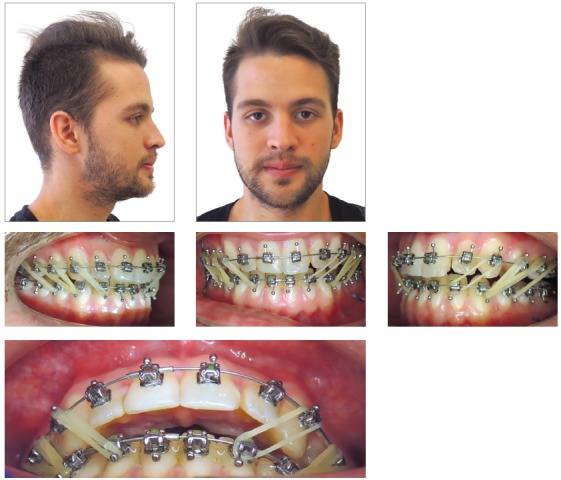
Class III intermaxillary elastics.

**Figure 8:**
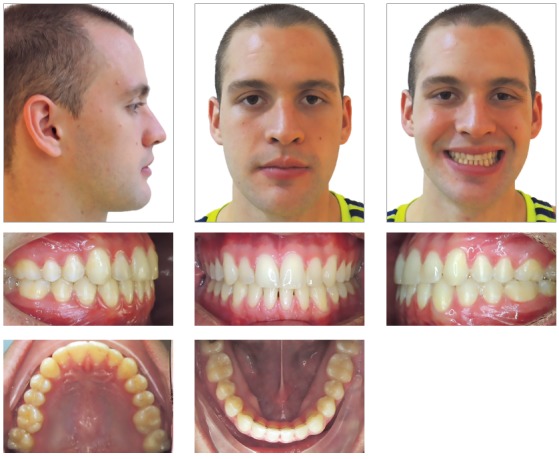
Post-treatment photographs.

**Figure 9:**
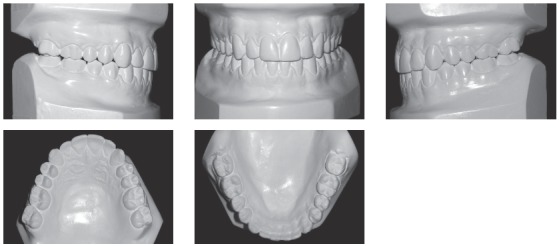
Post-treatment dental casts.

**Figure 10:**
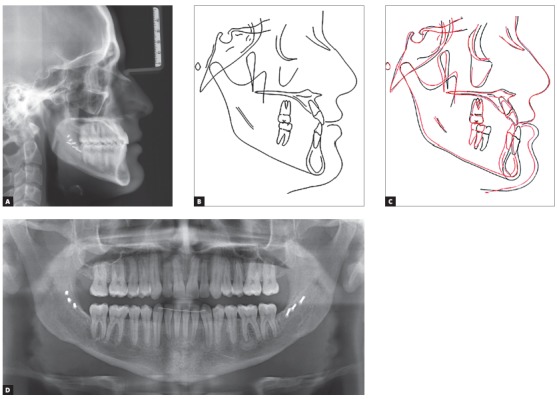
A) Post-treatment lateral cephalometric radiograph. B) Post-treatment cephalometric tracing. C) Superimposition of pre and post-treatment cephalometric tracings. D) Post-treatment panoramic radiograph.

**Figure 11:**
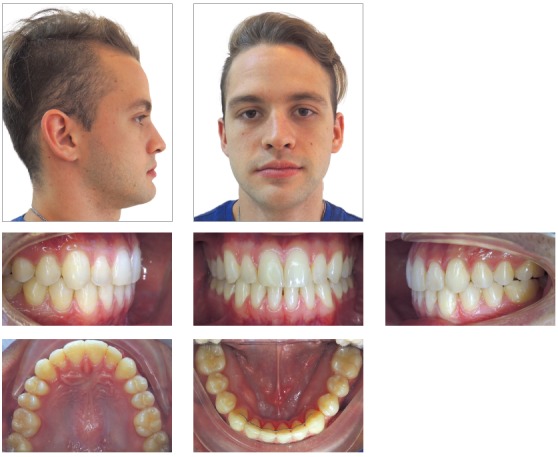
Follow-up photographs (24 months).

After 15 days, 1/8 3.5 oz intermaxillary elastics were used (Fig 7) and the archwires were changed to 0.014 x 0.025-in CuNiTi (Ormco Corporation, Orange, CA). One month after surgery 0.018 x 0.025-in CuNiTi archwires (Ormco Corporation, Orange, CA) were placed and Class III intermaxillary elastics were continued. Then, 0.019 x 0.025-in TMA arches (Ormco Corporation, Orange, CA) were placed six weeks later. The orthodontic treatment was completed five months after mandibular setback, showing great improvements in facial profile, Class I occlusion with ideal overjet and overbite (Figs 8, 9, and 10). The 24-month posttreatment photographs show excellent stability of the treatment results (Fig 11). 

## DISCUSSION

Surgery-first approach (SFA) was first proposed by Nagasaka et al,^15^ in 2009. With the orthognathic surgery performed before the orthodontic correction, total treatment time could be reduced to even less than the average period for presurgical orthodontics.^16^ ^-^ ^19^ Considering the number of patients who want orthognathic surgery mainly for esthetic reasons and would appreciate a shorter treatment time, SFA offers an attractive alternative for managing skeletal malocclusions while improving patients’ self-esteem and function at the beginning of treatment.^20^ ^,^ ^21^

The authors described several advantages offered by the surgery-first approach: (1) Improvement in patient’s facial esthetics and dental function in early treatment, rather than following a possible period of years, (2) improvement in patient’s swallowing and speech functions after surgery, (3) the proceeding of orthodontic tooth movement at a much faster pace following surgery, thus reducing the overall treatment time, (4) improved cooperation of the patient during orthodontic treatment, (5) easier orthodontic tooth movement following restoration of the normal functional and anatomic relationships of the bony skeleton and surrounding soft tissues; and (6) stability of results equal to, or in some cases superior to, those achieved using the traditional orthodontics-first approach.^22^

Most articles recommended that orthodontic appliances should be placed prior to surgery, even when using a surgery-first approach. Studies reported bonding the orthodontic brackets immediately before,^15^ ^,^ ^23^ 1 week before,^24-26^ 1 month before^27^ ^-^ ^29^ or 1-2 months before^30^ surgery. Only one of the papers reported the total elimination of preoperative orthodontic treatment and the fitting of orthodontic brackets 10-14 days after surgery^20^ Studies described that active orthodontic force can be applied before^26^ ^-^ ^29^ or shortly after^15^ ^,^ ^23^ ^-^ ^25^ ^,^ ^30^ surgery. Preoperative orthodontic preparation can, therefore, be started immediately before or approximately 1-2 months before surgery. Occasionally, it might be completely eliminated. 

The shortest reported treatment time for postoperative orthodontic treatment was 4 months for correction of a skeletal Class III malocclusion with anterior open bite and dental crowding^26^ and 4.5 months in the management of unilateral condylar hyperplasia,^11^ similar to this case report, with total treatment time of 5 months. Most studies described completing postoperative orthodontic treatment within approximately 1 year^15^ ^,^ ^27^ ^,^ ^28^ ^,^ ^30^ or in 6-9 months.^20^ ^,^ ^23^ ^,^ ^25^ Treatment time was approximately 6-12 months shorter using the SFA, compared to using a conventional orthodontics-first approach. Only one study described similar treatment time (approximately 1.5 years) for both approaches.^29^

There is no doubt that SFA requires precise and accurate diagnosis and planning. Post-surgical orthodontic movements must be carefully executed according to the surgical plan, which implies constant communication between orthodontist and oral surgeon. 

To expedite post-surgical orthodontics, Insignia System (Ormco Corporation, Orange, CA) is an important tool for offering customized brackets and archwires, also diminishing errors from appliance positioning. Customized devices in orthodontics have been reported before. Subjects treated with SureSmile (OraMetrix, Richardson, Tex) were compared with those undergoing conventional orthodontic treatment, concluding that treatment time was 7 months shorter in patients treated with SureSmile.^31^ Saxe et al^32^ obtained comparable results. However, SureSmile technology (OraMetrix, Richardson, Tex) customizes only the archwires, using robotically-assisted archwire bending technology.^32^ ^,^ ^33^ Insignia (Ormco Corporation, Orange County, CA) customizes bracket prescription, bonding and archwires.^14^ Besides, the light forces produced by the passive self-ligating system with high-tech archwires will control the transverse dimension in coordination with post-surgical sagittal changes.^19^

With two-dimensional (2D) imaging, the most usual problems are landmark identification, image distortion and magnification.^34^ ^,^ ^35^ However 2D imaging remains as the gold standard for the craniofacial region. The 3D computer-assisted surgical planning benefits the specialists because it can predict surgical movements including translations in anteroposterior, lateral, and vertical directions, and rotations around the x-, y-, and z-axes, the so-called pitch, roll, and yaw rotations^36^ and this is an undisputed advantage in determining the best treatment option. 

## CONCLUSIONS

» The 3D diagnostics, digital surgical planning and CAD/CAM customized bracket systems with passive self-ligation offer a more accurate alternative to improve the efficiency of orthodontic-surgical treatment.

» SFA helps to reduce treatment time, delivering aesthetic results from the beginning, which generates greater acceptance in surgical patients.
